# Fermentation of Xylose Causes Inefficient Metabolic State Due to Carbon/Energy Starvation and Reduced Glycolytic Flux in Recombinant Industrial *Saccharomyces cerevisiae*

**DOI:** 10.1371/journal.pone.0069005

**Published:** 2013-07-09

**Authors:** Akinori Matsushika, Atsushi Nagashima, Tetsuya Goshima, Tamotsu Hoshino

**Affiliations:** 1 Biomass Refinery Research Center, National Institute of Advanced Industrial Science and Technology, Hiroshima, Japan; 2 Human Metabolome Technologies, Inc., Yamagata, Japan; 3 Graduated School of Life Science, Hokkaido University, Hokkaido, Japan; University of Nottingham, United Kingdom

## Abstract

In the present study, comprehensive, quantitative metabolome analysis was carried out on the recombinant glucose/xylose-cofermenting *S*. *cerevisiae* strain MA-R4 during fermentation with different carbon sources, including glucose, xylose, or glucose/xylose mixtures. Capillary electrophoresis time-of-flight mass spectrometry was used to determine the intracellular pools of metabolites from the central carbon pathways, energy metabolism pathways, and the levels of twenty amino acids. When xylose instead of glucose was metabolized by MA-R4, glycolytic metabolites including 3- phosphoglycerate, 2- phosphoglycerate, phosphoenolpyruvate, and pyruvate were dramatically reduced, while conversely, most pentose phosphate pathway metabolites such as sedoheptulose 7- phosphate and ribulose 5-phosphate were greatly increased. These results suggest that the low metabolic activity of glycolysis and the pool of pentose phosphate pathway intermediates are potential limiting factors in xylose utilization. It was further demonstrated that during xylose fermentation, about half of the twenty amino acids declined, and the adenylate/guanylate energy charge was impacted due to markedly decreased adenosine triphosphate/adenosine monophosphate and guanosine triphosphate/guanosine monophosphate ratios, implying that the fermentation of xylose leads to an inefficient metabolic state where the biosynthetic capabilities and energy balance are severely impaired. In addition, fermentation with xylose alone drastically increased the level of citrate in the tricarboxylic acid cycle and increased the aromatic amino acids tryptophan and tyrosine, strongly supporting the view that carbon starvation was induced. Interestingly, fermentation with xylose alone also increased the synthesis of the polyamine spermidine and its precursor *S*-adenosylmethionine. Thus, differences in carbon substrates, including glucose and xylose in the fermentation medium, strongly influenced the dynamic metabolism of MA-R4. These results provide a metabolic explanation for the low ethanol productivity on xylose compared to glucose.

## Introduction

The yeast *Saccharomyces cerevisiae* has long been used in the food and beverage industry due to its high growth rate, rapid fermentation rate, and high ethanol productivity under anaerobic conditions. These characteristics, together with high tolerance for ethanol and low pH, make it one of the most robust and effective ethanol-producing organisms in industrial processes. Accordingly, *S*. *cerevisiae* is now widely used for the production of biofuels and chemicals [1]. The transition of raw materials and energy production from fossil to renewable resources will be aided by the efficient utilization of lignocellulosic biomass generated in the agricultural and forestry sectors. This has the potential to provide a wide variety of social, economic, and environmental benefits. This renewable raw material contains carbohydrates such as cellulose and hemicellulose that can be used to produce ethanol by fermentation [2]. Xylose, the most abundant pentose sugar in hemicellulose, makes up a sizable fraction of lignocellulosic hydrolysates [3], and complete conversion of xylose to ethanol is therefore necessary to make the biomass-to-ethanol process economical [4].

*S*. *cerevisiae* cannot naturally utilize xylose for growth or fermentation. On the other hand, xylulose, an isomerization product of xylose, can be metabolized through the non-oxidative pentose phosphate pathway (PPP) and the glycolysis pathway. Therefore, this yeast has been extensively engineered to acquire a xylose metabolic pathway converting xylose to xylulose as reported in several recent reviews [5], [6], [7], [8]. The construction of an efficient metabolic pathway for xylose fermentation in *S*. *cerevisiae* has been approached via the introduction of one of two heterologous pathways [9]: the oxido-reductive pathway with xylose reductase (XR) and xylitol dehydrogenase (XDH), or the isomerization pathway with xylose isomerase (XI). In the oxido-reductive pathway found in fungi, xylose is first reduced to xylitol by NAD(P)H-dependent XR, and then xylitol is oxidized to xylulose by NAD^+^-dependent XDH. On the other hand, cofactor-independent XI directly converts xylose to xylulose by the isomerization pathway mainly found in bacteria. Heterologous expression of XI in *S*. *cerevisiae* does not cause an intracellular redox imbalance during xylose fermentation; however, almost all XI activities are too low to enable anaerobic growth on xylose, and the rate of xylose consumption is much lower in the XI-expressing *S*. *cerevisiae* strains than in the XR- and XDH-expressing strains [9], [10].

In addition to the introduction of xylose-to-xylulose conversion pathways in *S*. *cerevisiae*, further genetic engineering and evolutionary engineering, including overexpression of the endogenous xylulokinase (XK), changes in the intracellular redox balance between the XR and XDH reactions, engineering of xylose transport, and potentiation of the pentose phosphate pathway, have been used to improve xylose fermentation [5], [6], [7], [8]. Although several xylose-utilizing *S*. *cerevisiae* strains have been successfully engineered and are able to ferment xylose into ethanol, the rate and yield of ethanol production from xylose in these strains are very low compared to glucose fermentation, and thus some fundamental barriers remain to their commercial and industrial bio-process use. Therefore, in combination with targeted metabolic and evolutionary engineering approaches, it is necessary to understand the molecular mechanisms of xylose utilization in engineered *S*. *cerevisiae* strains.

Multi-omics analyses, including those of the genome, transcriptome, proteome, metabolome, and fluxome, are promising tools both for the characterization of bioprocesses and for the design of novel strategies to enhance the production of ethanol [11], [12]. Metabolomics is an emerging analytical technique for systemic and global determination of metabolite profiles, which is useful for a better understanding of physiology. Metabolites as well as proteins are directly involved in cellular biochemistry and thereby closely dictate the physiology of an organism [13]. Moreover, metabolomics has led to the identification of a bottleneck reaction that reduces the rate of carbohydrate consumption during the production of useful products in *S*. *cerevisiae* [14]. Accordingly, a metabolomics approach can be used to determine the rate-limiting reactions in xylose-utilizing *S*. *cerevisiae* and therefore target metabolic changes to enhance the rate and yield of ethanol production [6]. However, very little is known about the response of targeted metabolites in xylose-fermenting *S*. *cerevisiae* strains when the carbon and energy sources are changed. Furthermore, a comprehensive quantitative description of the metabolic response to glucose-xylose cofermentation and fermentation of xylose alone is currently not available, raising the question of how metabolism changes in response to these different carbon sources under anaerobic conditions. Therefore, the metabolic basis for differences in ethanol fermentation efficiency from xylose or mixed xylose and glucose compared to glucose remains elusive.

In the present study, using capillary electrophoresis time-of-flight mass spectrometry (CE-TOFMS) [15], [16], [17], a novel strategy for analyzing and differentially displaying metabolic profiles [18], we determined the effects of different carbon sources containing glucose, xylose, or their mixtures on fermentation using the recombinant flocculent *S*. *cerevisiae* strain MA-R4 [19]. This strain was previously engineered for xylose metabolism by overexpressing XR and XDH from *Scheffersomyces* (*Pichia*) *stipitis*, and XK from *S*. *cerevisiae*, using the effective xylulose-utilizing flocculent *S*. *cerevisiae* strain IR-2 [20]. The major advantages of CE-TOFMS include its extremely high resolution, high throughput, and ability to simultaneously quantify all charged low molecular weight compounds in a sample. Novel metabolomic responses to different substrates presented in this study were analyzed with quantitative data obtained for 108 main metabolites, including key intermediates in PPP, glycolysis, and the tricarboxylic acid (TCA) cycle, of MA-R4. The results provide new insights into the carbon and energy metabolism of xylose-fermenting recombinant *S*. *cerevisiae*.

## Materials and Methods

### Yeast Strains and Media

The recombinant xylose-utilizing *S*. *cerevisiae* strain MA-R4, derived from the alcohol-fermenting diploid and flocculent yeast strain IR-2, was used in this study. MA-R4 was genetically engineered with the chromosome-integrated *XYL1* and *XYL2* genes that encode XR and XDH from *S*. *stipitis,* along with the endogenous *XKS1* gene that encodes XK under the control of the *PGK* promoter [19], [20]. For the construction of strain MA-R4, plasmid pAUR-XKXDHXR [21] was digested with the restriction enzyme *Bsi*WI and chromosomally integrated into the *aur1* locus of IR-2. MA-R4 was maintained by selective growth on yeast peptone (YP) medium (10 g/L yeast extract and 20 g/L peptone) supplemented with 20 g/L glucose in the presence of 0.5 mg/L aureobasidin A (Takara Bio, Kyoto, Japan). Glucose (45 g/L) was added to YP medium to produce YPD medium. Xylose (45 g/L) was added to YP medium to produce YPX medium. The addition of glucose (45 g/L) and xylose (45 g/L) to YP medium produced YPM medium. The YPD, YPM, and YPX media were used as the anaerobic fermentation media in this study.

### Fermentation

For anaerobic batch fermentation, MA-R4 was first cultivated aerobically in 5 mL YP medium supplemented with 20 g/L glucose and 0.5 mg/L aureobasidin A for 36 h at 30°C. The resulting culture was centrifuged at 6000×*g* for 5 min at 4°C, and the pelleted cells were washed and resuspended in distilled water. The washed cells were inoculated into 20 mL fermentation medium (YPD, YPM, and YPX). For all fermentation media, the initial cell density was adjusted to approximately 4.12 g (dry cell weight (DCW)) per liter. Anaerobic batch fermentations were performed at 30°C in 50-mL sterilized closed bottles with magnetic stirring as described previously [19], [20], [21]. Samples (0.3 mL) of fermentation broth (YPD, YPM, and YPX) were taken at specified intervals and diluted 4-fold with 8 mM H_2_SO_4_. These diluted samples were stored at −30°C for high-performance liquid chromatography (HPLC) analysis of substrates and fermentation products. All experiments were performed in triplicate.

### Quantification of Biomass, Substrates, and Fermentation Products

DCW was determined using a UV-2450 spectrophotometer (Shimadzu, Kyoto, Japan) to measure the absorbance of the samples at 600 nm, as described previously [21]. Concentrations of glucose, xylose, ethanol, xylitol, glycerol, and acetic acid were determined with an HPLC apparatus (Jasco, Tokyo, Japan) equipped with a refractive index detector (RI-2031Plus; Jasco) using an Aminex HPX-87H (Bio-Rad Laboratories, Hercules, CA, USA) and Cation H Refill Guard (Bio-Rad) column. The HPLC apparatus was operated at 65°C, with 5 mM H_2_SO_4_ as the mobile phase, a flow rate of 0.6 mL/min, and an injection volume of 20 µL.

### Extraction of Intracellular Metabolites

Intracellular metabolites were extracted as described previously with some modifications [22]. Briefly, MA-R4 cells grown as described above were harvested for metabolome analysis at times indicated in the Results section below. Triplicate samples were collected at each time point (all eight time points). Cultures including approximately 10^9^ cells (calculated as optical density at 600 nm×sampling volume of culture (mL) = 20) were filtered by a suction-filtering system using a 0.4 µm pore size filter. The residual cells on the filter were washed twice with 10 mL of Milli-Q water. The filter was immersed in 2 mL of methanol including 5 µM each of internal standard reagents onto a plastic dish. The dish was sonicated for 30 sec using a Branson 2510 ultrasonic syringe (not an ultrasonic cell disrupter) (Branson Ultrasonics, Danbury, CT, USA) to suspend the cells completely. A 1.6 mL portion of the methanol cell suspension was transferred to a Falcon Blue Max Jr., 352097 centrifugal tube (15 mL) (Becton, Dickinson & Co. NJ, USA) and mixed with 1.6 mL of chloroform and 640 µl of Milli-Q water. After vortexing thoroughly, the mixture was centrifuged at 2,300×*g* for 5 min at 4°C. The aqueous layer (375 µL) was distributed to four Amicon Ultrafree-MC ultrafilter tips (Millipore, Billerica, MA, USA) and centrifuged at 9,100×*g* for 120 min at 4°C. The filtrate was dried and dissolved in 50 µL of Milli-Q water.

### Metabolites Analysis and Data Processing

CE-TOFMS analysis was performed using the Agilent CE-TOFMS system (Agilent, Palo Alto, CA, USA) as described previously [15], [16], [17], [22], [23] with slight modifications. Cationic metabolites were separated through a fused silica capillary (50 µm internal diameter×80 cm total length) preconditioned with a commercial buffer (H3301-1001, Human Metabolome Technologies (HMT), Tsuruoka, Japan), and a commercial sheath liquid (H3301-1020, HMT) was delivered. Sample solutions were injected at a pressure of 50 mbar for 10 sec. The applied voltage was set at 27 kV. Electrospray ionization-mass spectrometry (ESI-MS) was conducted in the positive-ion mode and the capillary voltage was set at 4,000 V. Exact mass data were acquired over a 50–1,000 *m/z* range. Anionic metabolites were analyzed also through a fused silica capillary preconditioned with a commercial buffer (H3302-1021 and H3302-1022, HMT), and the aforementioned sheath liquid was delivered. Sample solutions were injected at a pressure of 50 mbar for 25 sec. The applied voltage was set at 30 kV. ESI-MS was conducted in the negative mode, and the capillary voltage was set at 3,500 V. The scanning condition of the spectrometer was configured in the same manner as the cationic metabolite analysis. Peak extraction was carried out with the automatic integration software MasterHands [24]. Each metabolite was identified and quantified based on the peak information, including *m/z*, migration time, and peak area. The quantified data were then evaluated for statistical significance. Principal component analysis (PCA) and hierarchical clustering analysis (HCA) were processed by SampleStat (HMT) and PeakStat (HMT), respectively.

## Results and Discussion

### Ethanol Fermentation by a Xylose-fermenting Industrial Yeast

**Figure 1:**
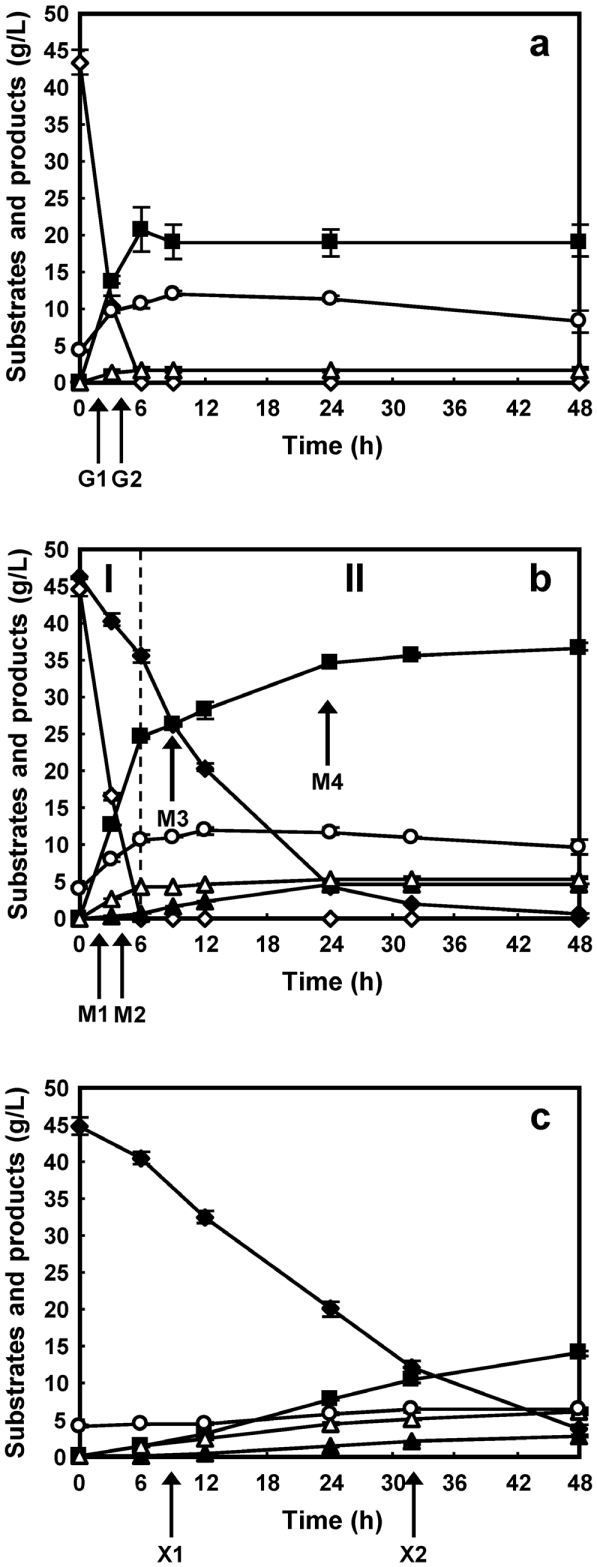
Time-dependent ethanol fermentation profiles of recombinant *S. cerevisiae* strain MA-R4 in YPD medium containing 45 g/L glucose (a), YPM medium containing 45 g/L glucose and 45 g/L xylose (b), and YPX medium containing 45 g/L xylose (c). Symbols: *open diamonds*, glucose; *closed diamonds*, xylose; *closed squares*, ethanol; *closed triangles*, xylitol; *open triangles*, glycerol; *open circles*, dry cell weight. Small amounts of acetic acid (less than 1.5 g/L) were detected (data not shown). Error bars indicate standard error (SE). Values are the means of three independent experiments. In the mixed sugar fermentation, two phases, the glucose/xylose co-consumption phase (phase I) and the xylose consumption phase (phase II), are indicated. The arrows (denoted by G1, G2, M1, M2, M3, M4, X1, and X2) indicate stages at which samples were taken for metabolomics analyses.

To determine the effect of different carbon and energy sources on ethanol fermentation by the recombinant industrial *S*. *cerevisiae* strain MA-R4, fermentation was performed anaerobically in YP-based media supplied with 45 g/L glucose (YPD medium), 45 g/L glucose and 45 g/L xylose (YPM medium), and 45 g/L xylose (YPX medium). As shown in Figure 1a–c, MA-R4 showed different fermentation patterns depending on the carbon source of the medium. MA-R4 rapidly fermented glucose in both YPD and YPM media within 6 h (Figure 1a and b). In contrast, xylose was more slowly fermented by MA-R4 than glucose, which fermented almost all the xylose in both YPX and YPM media within 48 h (Figure 1c and b). In fermentation from mixed sugar (YPM medium), MA-R4 was able to simultaneously co-ferment glucose and xylose (0 to 6 h; Figure 1b), although glucose was utilized more rapidly than xylose. The co-fermentation performance of MA-R4 is consistent with our previous findings [19], [25], [26], [27]. As schematically shown in Figure 1b, we can distinguish the glucose/xylose co-consumption phase (phase I) from the xylose-only consumption phase (phase II). The specific glucose consumption rate (1.135 g-glucose/g-DCW/h) of MA-R4 during fermentation with glucose as the sole carbon source (YPD medium) was nearly identical to that (1.173 g-glucose/g-DCW/h) during phase I in fermentation of the glucose/xylose mixture (YPM medium) (Table 1). However, the specific xylose consumption rate of MA-R4 was slightly lower in fermentation with YPX medium (0.180 g-xylose/g-DCW/h) than in phase II in fermentation with YPM medium (0.226 g-xylose/g-DCW/h) (Table 1). MA-R4 grew quickly during both glucose fermentation (0 to 6 h; Figure 1a) and glucose/xylose cofermentation (0 to 6 h; Figure 1b), but grew slowly or scarcely at all during the xylose-only fermentation (6 to 48 h; Figure 1b and 0 to 48 h; Figure 1c). In fermentation using YPD and YPM media, the initial cell concentrations (approximately 4.12 g/L) increased after 6 h of fermentation to 10.6 and 10.8 g/L, respectively (Figure 1a and b). Meanwhile, the cell concentrations after 48 h of xylose-only fermentation using YPM and YPX media were 9.65 and 6.46 g/L, respectively (Figure 1b and c).

**Table 1:** Summary of 48-h fermentations in different media by *S. cerevisiae* strain MA-R4.

Medium	Maximum ethanol concentration (g/L)	Specific glucose consumption rate(g-glucose/g-DCW/h)	Specific xylose consumption rate(g-xylose/g-DCW/h)	Specific ethanol production rate(g-ethanol/g-DCW/h)	Ethanol yield (g-ethanol/g-consumed total sugar)	Xylitol yield (g-xylitol/g-consumed xylose)	Glycerol yield (g-glycerol/g-consumed total sugar)	Acetic acid yield(g-acetic acid/g-consumed total sugar)
YPD	20.7±2.9	1.135±0.026	ND	0.475±0.006	0.478±0.015	ND	0.042±0.002	0.011±0.001
YPM	36.8±0.5	1.173±0.046	0.226±0.047	0.372±0.019	0.404±0.002	0.101±0.003	0.060±0.001	0.016±0.000
YPX	14.4±0.2	ND	0.180±0.011	0.051±0.002	0.343±0.014	0.068±0.005	0.143±0.009	0.021±0.001

Values are the averages of three independent experiments ± standard deviation.

ND, not detectable.

MA-R4 produced a maximum amount of ethanol of 20.7 g/L after 6 h of fermentation with YPD medium (Table 1 and Figure 1a), whereas at the end of the 48-h fermentation, the highest ethanol concentration produced by MA-R4 with YPM and YPX media was 36.8 g/L (Table 1 and Figure 1b) and 14.1 g/L (Table 1 and Figure 1c), respectively. In fermentations using mixtures of glucose and xylose (YPM medium), ethanol was quickly produced during the glucose/xylose co-consumption phase (phase I; Figure 1b), whereas ethanol was gradually produced during the xylose-only consumption phase (phase II; Figure 1b). The rate of specific ethanol production achieved in fermentation with YPX (0.051 g-ethanol/g-DCW/h) was approximately 89% lower than that with YPD (0.475 g-ethanol/g-DCW/h) (Table 1), which is consistent with our previous results from fermentation with glucose or xylose alone (90 g/L total sugar) [27]. The ethanol yield, which was estimated as grams of ethanol produced per gram of total sugar consumed (g/g) after 48 h of fermentation, of MA-R4 was 0.478 g/g (93.7% of the theoretical yield), 0.404 g/g (79.2%), and 0.343 g/g (67.3%) in YPD, YPM, and YPX, respectively (Table 1). Thus, ethanol yield decreased as the proportion of xylose increased. A lower ethanol yield at high proportions of xylose was also observed in our previous study [27]. For all media, a small amount of glycerol (no more than 5.88 g/L) and a small amount of acetic acid (no more than 1.43 g/L) was produced, both of which were at higher concentrations in the presence of xylose (i.e., YPM and YPX media) than in the absence of xylose (i.e., YPD medium) (Figure 1a–c). Xylitol production was maintained below 4.71 g/L during fermentation using YPM and YPX media (Figure 1b and c). As shown in Table 1, the xylitol yields were 0.101 and 0.068 g-xylitol/g-consumed xylose in fermentation with YPM and YPX media, respectively. Meanwhile, the glycerol and acetic acid yields increased with an increasing proportion of xylose (Table 1). The higher glycerol and acetic acid yields in YPX may be directly related to the low ethanol yield. These results are fully compatible with the idea that ethanol selectivity is reduced when a higher percentage of xylose is present.

### Target Metabolites of CE-TOFMS Based Metabolomics

The metabolic response to changes in carbon sources, including glucose, xylose, and mixed sugars of glucose and xylose, was determined by measuring intracellular metabolites in MA-R4 at specific (eight total) time points during 48 h of fermentation. In fermentation with glucose or xylose as the sole carbon source, samples of the yeast cells were harvested at each of two time points, while samples were collected at four time points in mixed-sugar fermentation, in which samples were taken at two time points during phase I (glucose/xylose co-consumption phase) and phase II (xylose-only consumption phase), respectively. Representative time-courses are displayed in Figure 1. After the initiation of glucose fermentation using YPD medium, samples of MA-R4 were harvested at 2 h and 4.5 h, which were denoted as G1 and G2 stages, respectively (Figure 1a). During phase I in mixed sugar fermentation with YPM medium, samples were collected at 2 h and 4.5 h (denoted as M1 and M2 stages in Figure 1b, respectively), whereas samples were taken at 9 h and 24 h (denoted as M3 and M4 stages in Figure 1b, respectively) during phase II. Samples were harvested at 9 h and 32 h (denoted as X1 and X2 stages in Figure 1c, respectively) from the start of the cultures containing xylose alone (YPX medium). These sampling time points were classified into two phases, those that mainly fermented glucose (G1, G2, M1, and M2 stages; glucose fermentation phases) and those that exclusively fermented xylose (M3, M4, X1, and X2 stages; xylose fermentation phases). After the sampling, intracellular metabolites were extracted, and then the ionic metabolites were measured by CE-TOFMS as described in the Materials and Methods section above. As a result, a total of 255 metabolites containing 154 cationic and 101 anionic metabolites were identified. Of these, 108 metabolites, which include amino acids, organic acids, sugar phosphates, and nucleotides [17], were quantified using external standards, and targeted metabolites analysis was performed.

### Changes in Central Carbon Metabolism

**Figure 2:**
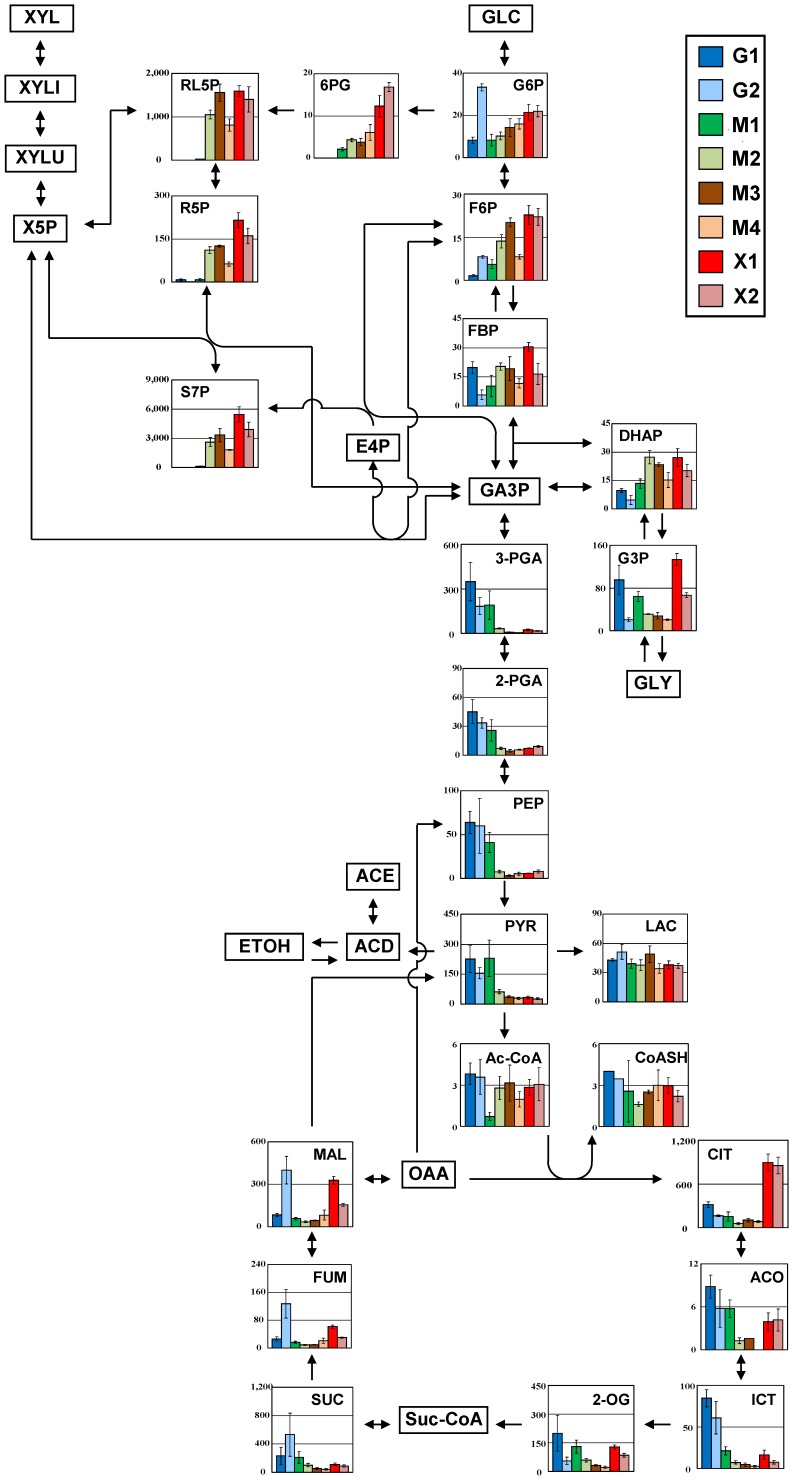
Comparison of the metabolites in central carbon metabolism (including glycolysis, the pentose phosphate pathway, and tricarboxylic acid cycle pathway) of MA-R4 during the fermentation of glucose alone (G1 and G2 stages), mixed sugars of glucose and xylose (M1, M2, M3, and M4 stages), and xylose alone (X1 and X2 stages). Each bar represents the mean amounts of the metabolites (pmol/OD600⋅mL) in each sampling stage (G1, G2, M1, M2, M3, M4, X1, and X2). These eight sampling points during the fermentation experiments are shown in Figure 1. Error bars represents the standard deviations from three independent experiments. The arrowheads in the figure represent the direction of enzymatic reactions. Abbreviations: GLC, glucose; XYL, xylose; XYLI, xylitol; XYLU, xylulose; X5P, xylulose 5-phosphate; G6P, glucose 6-phosphate; F6P, fructose 6-phosphate; FBP, fructose 1,6−bisphosphate; GA3P, glyceraldehyde 3-phosphate; DHAP, dihydroxyacetone phosphate; G3P, glycerol 3-phosphate; GLY, glycerol; 6PG, 6-phosphogluconate; RL5P, ribulose 5-phosphate; R5P, ribose 5-phosphate; S7P, sedoheptulose 7- phosphate; E4P, erythrose 4-phosphate; 3-PGA, 3- phosphoglycerate; 2-PGA, 2- phosphoglycerate; PEP, phosphoenolpyruvate; PYR, pyruvate; LAC, lactate; ACD, acetaldehyde; ACE, acetate; ETOH, ethanol; Ac-CoA, acetyl coenzyme A; CoASH, coenzyme A; CIT, citrate; ACO, aconitate; ICT, isocitrate; 2-OG, 2-oxoglutarate; Suc-CoA, succinyl coenzyme A; SUC, succinate; FUM, fumarate; MAL, malate; OAA, oxaloacetate.

Figure 2 shows changes in the amounts of the metabolites related to central carbon metabolism pathways such as glycolysis, PPP, and the TCA cycle. Among the 108 metabolites quantified, glyceraldehyde 3-phosphate (GA3P) and erythrose 4-phosphate (E4P) in central carbon metabolism were not detected. The most drastic changes in response to different carbon sources were observed in PPP and glycolysis. As shown in Figure 2, the amounts of 6-phosphogluconate (6PG), ribulose 5-phosphate (RL5P), ribose 5-phosphate (R5P), and sedoheptulose 7- phosphate (S7P) in the PPP were greatly increased during xylose fermentation phases (M3, M4, X1, and X2 stages), while the amounts of these metabolites scarcely accumulated in fermentation with glucose alone (G1 and G2 stages). In particular, 6PG was highly increased by more than 2.0-fold in fermentation with xylose alone (X1 and X2 stages) compared to other fermentation phases. These findings are not consistent with those obtained using another xylose-utilizing yeast; it has been reported that most metabolites of the PPP were hardly affected [28].

Products of the first half of glycolysis, from glucose 6-phosphate (G6P) to fructose 1,6-bisphosphate (FBP) and dihydroxyacetone phosphate (DHAP), accumulated at a relatively high amount in xylose fermentation phases in concert with the intermediates in the PPP, except for the high level of G6P in G2. On the other hand, products of the last half of glycolysis, from 3-phosphoglycerate (3-PGA) to pyruvate (PYR), showed the opposite of the first half of glycolysis. In other words, 3-PGA, 2- phosphoglycerate (2-PGA), phosphoenolpyruvate (PEP), and PYR in the downstream portion of the glycolytic pathway accumulated at very low levels during xylose fermentation phases (M3, M4, X1, and X2 stages). For instance, in these phases the levels of PEP decreased to 13% of that of glucose alone. These results are consistent with previous findings that flux through glycolysis is reduced (∼27-fold) when xylose replaces glucose as a substrate [28]. Taken together, these findings suggest that the significant decreases in the glycolytic intermediates upstream of PYR during xylose fermentation phases are most likely due to the accumulation of intermediates in PPP. Therefore, it is necessary to redirect and increase the metabolic flux through glycolysis from the PPP to improve xylose fermentation by MA-R4.

The amount of acetyl coenzyme A (Ac-CoA) did not differ largely between glucose and xylose fermentation phases, although in the M1 stage the amount of this metabolite declined remarkably. There were no differences in the amount of lactate (LAC) in any stage of fermentation. Meanwhile, glycerol 3-phosphate (G3P) substantially accumulated in the initial phase of fermentation with xylose alone (X1 stage), which may be associated with an increase in the formation of glycerol in YPX medium as mentioned above. The behavior of TCA cycle intermediates was more complicated than those of PPP and glycolysis. The concentrations of citrate (CIT) increased in fermentation with xylose alone (X1 and X2 stages), reaching more than 2.7-fold higher compared with glucose fermentation phases. Interestingly, Redon et al. [29] found that the citrate utilization pathway in *Lactococcus lactis* is activated during carbon starvation. In contrast, in the glucose fermentation phases (G1 and G2 stages), aconitate (ACO) and isocitrate (ICT) increased in concentration. Concentrations of three dicarboxylates, succinate (SUC), fumarate (FUM) and malate (MAL), increased during the late phase of fermentation with glucose alone (G2 stage).

### Changes in Amino Acids

Table 2 shows changes in the amounts of 20 amino acids. Evidently, a number of amino acids tended to decline in the xylose fermentation phases. These include serine (Ser), glycine (Gly), valine (Val), leucine (Leu), aspartate (Asp), lysine (Lys), threonine (Thr), isoleucine (Ile), and methionine (Met). Many of these amino acids were the most highly accumulated in fermentation with glucose alone (G1 and G2 stages). Of these amino acids, Met showed the characteristic metabolism variation pattern in response to different carbon sources, in which the amount of Met increased during the initial phase of glucose fermentation (G1 and M1 stages) by 5.6-fold. On the other hand, the aromatic amino acids, tryptophan (Trp) and tyrosine (Tyr), and precursors of glutamate (Glu), proline (Pro), and glutamine (Gln), were the most highly accumulated in the initial phase of fermentation with xylose alone (X1 stage). Increased accumulation of Trp and Tyr in the X1 stage was not anticipated, because these are most costly in regards to ATP required for their biosynthesis [30]. However, the result is consistent with previous reports [28] showing that levels of these aromatic amino acids are higher in yeast cells cultivated on xylose. Increased concentrations of the aromatic amino acids have been observed in yeast when starved for carbon under anaerobic conditions [28]. Similar results for alanine (Ala), asparagine (Asn), and Glu as well as the aromatic amino acids have also been reported for aerobic conditions [31]. Interestingly, the carbon starvation state appears to be the general response of the XI-expressing strain to xylose [32]. Considering our findings that the fermentation of xylose caused a dramatic increase of CIT in the TCA cycle and of the aromatic amino acids Trp and Tyr, we conclude that carbon and energy starvation conditions are common in MA-R4 during fermentation from xylose alone.

**Table 2:** The amounts of amino acids in each stage of fermentation by *S*. *cerevisiae* strain MA-R4.

	(pmol/OD600•mL)							
	G1	G2	M1	M2	M3	M4	X1	X2
Derived from 3-phosphoglycerate								
Ser	486±22	995±12	390±68	273±25	266±33	208±44	160±13	174±3
Gly	1373±89	1227±31	943±166	621±59	702±2	355±66	398±25	227±2
Cys	0.30±0.00	0.34±0.22	ND	0.15±0.04	0.10±0.00	0.24±0.10	0.23±0.08	0.36±0.24
Derived from phosphoenolpyruvate and erythrose 4-phosphate								
Trp	5.95±0.13	7.49±2.65	6.24±1.11	2.71±0.20	3.33±0.30	4.17±0.65	9.19±0.50	4.02±1.01
Phe	36.4±8.5	52.7±12.2	52.5±11.3	33.2±4.8	30.1±8.9	26.4±2.9	49.8±4.5	24.0±5.8
Tyr	34.0±4.5	35.1±7.6	32.1±6.8	15.7±2.0	13.0±1.8	13.5±1.8	71.2±8.8	15.3±2.8
Derived from pyruvate								
Ala	650±44	1541±80	505±89	914±62	921±84	738±165	691±41	731±48
Val	293±26	405±20	204±36	134±8	92±11	66±13	161±8	112±9
Leu	129±7	138±19	113±22	48±2	26±1	22±4	58±3	30±8
Derived from 2-oxoglutarate								
Glu	3138±76	2768±126	2661±560	1710±126	916±62	893±78	2389±229	1276±50
Gln	527±121	873±33	419±41	676±83	748±104	789±227	1279±105	733±6
His	1732±90	983±9	1207±266	557±35	428±65	482±124	1044±106	530±30
Pro	324±16	424±9	247±40	203±23	206±17	226±13	503±47	165±6
Arg	877±262	214±42	491±211	217±8	195±35	213±34	595±91	311±7
Derived from oxaloacetate								
Asp	557±132	1073±95	440±116	276±44	136±22	115±11	74±7	70±9
Asn	137±6	375±54	148±17	236±24	200±22	178±36	196±14	125±2
Lys	2403±611	1645±206	1394±517	1098±52	888±155	494±62	794±98	490±59
Thr	1035±39	861±41	631±113	247±24	169±10	84±15	228±13	93±4
Ile	136±13	130±11	97±23	38±4	25±2	19±2	33±2	22±2
Met	117±11	19±4	113±23	20±1	11±2	7.7±2	13±1	10±1

Values are the averages of three independent experiments ± standard deviation.

ND, not detectable.

### Changes in Nucleotides and Energy Charge

Table 3 shows the amounts of adenine and guanine nucleotides. Adenine and guanine pools were similarly affected when xylose instead of glucose was used as a substrate, supporting previous findings showing that guanine nucleotides are kept under tight control in correlation with adenine nucleotides [33]. The levels of the nucleotide triphosphates ATP and GTP, which are high-energy carrier molecules, were lower in the xylose fermentation phases, especially in M3 and M4 stages, than in the glucose fermentation phases. During fermentation with glucose alone (G1 and G2 stages), ATP substantially accumulated and increased by more than 4.5-fold compared to fermentation with xylose alone (X1 and X2 stages). In contrast to ATP and GTP, the concentrations of the nucleotide monophosphates AMP and GMP increased in xylose fermentation phases and decreased in glucose fermentation phases. The highest accumulation levels of AMP and GMP were observed during the late of phase II in mixed sugar fermentation (M4 stage), while the lowest levels of AMP and GMP were observed in the late phase of fermentation with glucose alone (G2 stage). The highest accumulation level of ADP was observed in the G1 stage, but the amount of ADP did not greatly differ between the remaining glucose and xylose fermentation phases. The level of GDP was slightly increased in the G1 stage, but in the remaining glucose fermentation phases (G2, M1, and M2 stages) the concentration of GDP had decreased by 2.5-fold compared to the xylose fermentation phases. As with the amounts of adenine and guanine nucleotides, the ratios of ATP/AMP and GTP/GMP also decreased in the xylose fermentation phases. The ATP/AMP ratios in the G1, G2, M1, M2, M3, M4, X1, and X2 stages were 5.39, 43.2, 4.44, 2.97, 0.33, 0.17, 1.99, and 0.73, respectively, while the GTP/GMP ratios in the G1, G2, M1, M2, M3, M4, X1, and X2 stages were 2.96, 15.9, 2.42, 2.91, 0.37, 0.21, 1.15, and 0.56, respectively. During glucose fermentation phases, the average ATP/AMP and GTP/GMP ratios were 14.0 and 6.05, respectively. Meanwhile, during xylose fermentation phases, the average ATP/AMP and GTP/GMP ratios were 0.81 and 0.57, respectively. Thus, the ATP/AMP and GTP/GMP ratios in the xylose fermentation phases were 17.3-fold and 10.6-fold higher than those in the glucose fermentation phases, respectively. A 10-fold decrease in the GTP/GMP ratio in response to a change from glucose to xylose fermentation has also been measured in recent studies [32], [34].

**Table 3:** The amounts of adenine and guanine nucleotides in each stage of fermentation by *S*. *cerevisiae* strain MA-R4.

	(pmol/OD600•mL)							
	G1	G2	M1	M2	M3	M4	X1	X2
ATP	180.1±32.7	276.6±13.6	70.2±41.8	43.0±2.4	15.6±3.6	10.9±3.6	40.1±9.4	25.4±15.5
ADP	72.5±25.7	28.3±6.6	21.2±11.7	18.3±2.7	30.4±6.8	29.8±4.3	27.2±5.1	27.5±2.6
AMP	33.4±9.3	6.4±0.9	15.8±4.4	14.5±1.1	47.0±9.8	64.2±17.8	20.2±1.4	34.6±12
GTP	67.3±12.4	47.7±3.9	28.6±15.6	26.2±2.8	13.4±3.7	11.0±3.2	33.8±6.8	20.9±14.9
GDP	36.5±9.1	13.4±1.8	12.6±6.9	16.6±3.1	29.2±7.9	31.3±5.1	30.3±3.8	27.9±1.3
GMP	22.7±4.3	3.0±0.2	11.8±4.7	9.0±1.5	35.8±7.0	53.3±6.1	29.3±2.6	37.0±12.1

Values are the averages of three independent experiments ± standard deviation.

**Figure 3:**
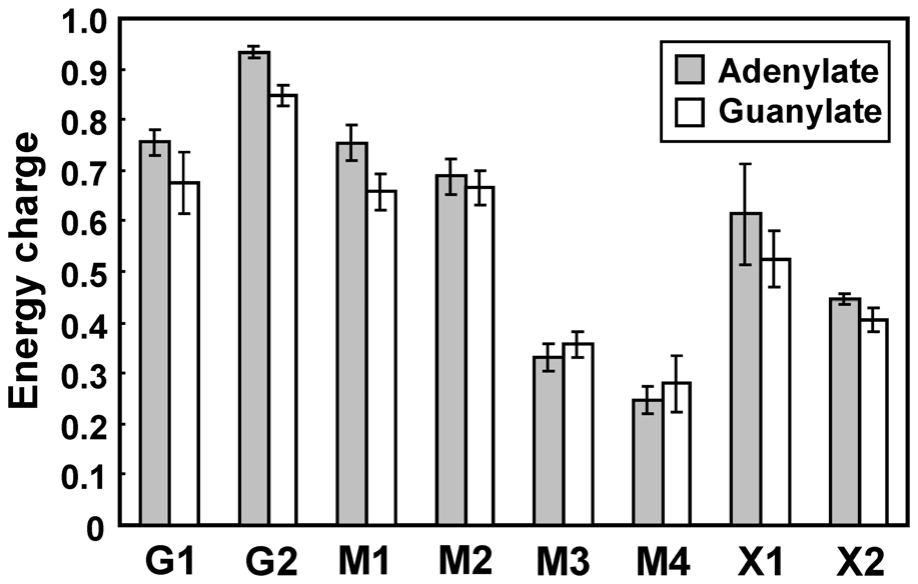
Adenylate and guanylate energy charges in MA-R4 during the fermentation of glucose alone (G1 and G2), mixed sugars of glucose and xylose (M1, M2, M3, and M4), and xylose alone (X1 and X2). The eight sampling stages during the fermentation experiments can be seen in Figure 1. The adenylate energy charge (gray bars) was calculated as follows: (ATP+½ADP)/(ATP+ADP+AMP). The guanylate energy charge (white bars) was calculated as follows: (GTP+½GDP)/(GTP+GDP+GMP). Error bars indicate standard error (SE). Values are averages from three independent experiments.

The amounts of adenine and guanine nucleotides were reflected in the adenylate energy charge (AEC) and guanylate energy charge (GEC) (Figure 3), which were calculated as follows: (([RTP] +0.5×[RDP])/([RTP]+[RDP]+[RMP]), R = A or G). The AEC has been suggested to be an important regulatory signal for controlling the energy balance and reflects the overall cellular energy status [35], [36]. Meanwhile, the GEC can be calculated analogously to the AEC, but it is not related to any biological process [32]. Both the AEC and GEC tended to increase during the glucose fermentation phases and decline during the xylose fermentation phases. The levels of the AEC and GEC were similar during the glucose fermentation phases (G1, M1, and M2 stages) and remained at approximately 0.73 and 0.67, respectively, although in the G2 stage the levels of the AEC and GEC were slightly increased to 0.93 and 0.85, respectively (Figure 3). The average level of the AEC (0.85) in the G1 and G2 stages agrees very well with previously reported values for *S*. *cerevisiae* growing on glucose [28], [32], [37], [38]. However, the average level of the AEC dropped by 38% in the X1 and X2 stages to 0.53. The average level of the GEC (0.76) in the G1 and G2 stages also resulted in a 38% reduction compared to that (0.47) in the X1 and X2 stages. The levels of the AEC and GEC were more severely affected during phase II in mixed sugar fermentation (M3 and M4 stages), in which the average levels of the AEC (0.29) and GEC (0.32) in M3 and M4 stages were 60% and 52% lower that those in the M1 and M2 stages, respectively. Thus, xylose fermentation by the MA-R4 strain leads to significant decreases in the AEC and GEC, particularly in the xylose-only consumption phase (phase II) of fermentation with mixed sugar. Reductions in the AEC and GEC during xylose fermentation phases were predominantly due to decreased levels of ATP and GTP and increased levels of AMP and GMP, yielding reduced ATP/AMP and GTP/GMP ratios (10.6- to 17.3-fold) as mentioned above. The energy state in MA-R4 during the xylose fermentation phases, reflected by lower AEC and GEC, was completely different from that of yeast cells exhibiting an active flux through glycolysis and instead resembled that of starving yeast cells [37], indicating that MA-R4 is energy limited when fermenting with xylose. Furthermore, the results from the energy charges revealed some metabolic differences between fermentation with xylose alone (X1 and X2 stages) and the phase II of mixed sugar fermentation (M3 and M4 stages).

### Changes in Polyamine Metabolism

**Figure 4:**
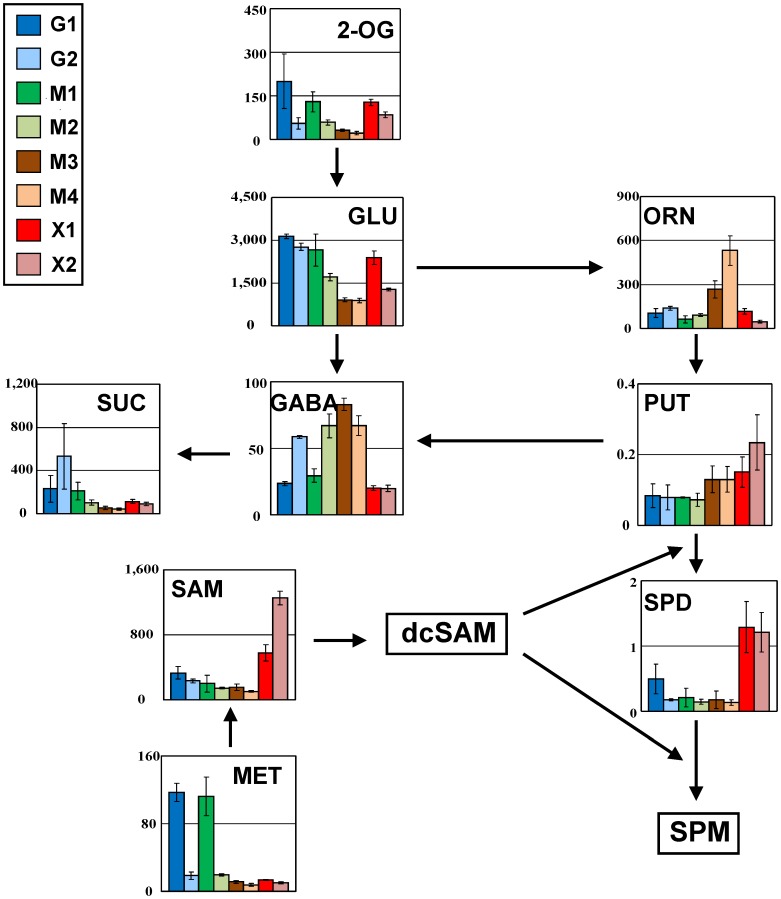
Comparison of the metabolites in the polyamine biosynthetic pathway of MA-R4 during the fermentation of glucose alone (G1 and G2 stages), mixed sugars of glucose and xylose (M1, M2, M3, and M4 stages), and xylose alone (X1 and X2 stages). Each bar represents the mean amounts of the metabolites (pmol/OD600⋅mL) in each sampling stage (G1, G2, M1, M2, M3, M4, X1, and X2) (see Figure 1). Error bars represents the standard deviations from three independent experiments. The arrowheads in the figure represent the direction of enzymatic reactions. Abbreviations: 2-OG, 2-oxoglutarate; GLU, glutamate; ORN, ornithine; PUT, putrescine; SPD, spermidine; SPM, spermine; GABA, γ-aminobutyric acid; SUC, succinate; MET, methionine; SAM, *S*-adenosylmethionine; dcSAM, decarboxylated *S*-adenosylmethionine.

The cellular polyamines putrescine (PUT), spermidine (SPD), and spermine (SPM) are ubiquitous compounds that are implicated in a wide range of growth and developmental processes in most organisms [39], [40]. Decarboxylated *S*-adenosylmethionine (SAM) is an essential precursor in the formation of both SPD and SPM (Figure 4). SAM is the major methyl group donor in many biological processes, and is a key regulatory compound in the biosynthesis of sulfur amino acids [41]. The metabolic analysis of MA-R4 also revealed that the different carbon sources caused several marked changes in polyamine metabolism (Figure 4). The fermentation of xylose as the sole carbon source led to a dramatic increase in the levels of SAM and SPD. SAM showed a 2.2-fold increase in the X2 stage compared to the X1 stage, whereas the level of MET was low in both X1 and X2 stages. In X1 and X2 stages, SPD accumulated to similarly high levels, exhibiting an approximately 3.6-fold increase compared with that in the G1 and G2 stages. In addition, the amount of PUT slightly increased in the X2 stage. Also of note, SPM could be quantified only when fermenting with xylose alone (0.12 and 0.10 pmol/OD600•mL in X1 and X2 stages, respectively), but in other fermentation phases, SPM was not detected probably due to very low levels. Taken together, these results clearly indicate that polyamines and their precursor SAM were specifically increased when fermenting with xylose alone. Meanwhile, ornithine (ORN), the key precursor for putrescine and polyamine biosynthesis, and γ-aminobutyric acid (GABA), which is produced via oxidation of putrescine, displayed more diverse accumulation patterns than polyamines and SAM.

A previous study has shown that high levels of SAM in yeast increase the growth in medium without sources of nitrogen and sulfur, and are recycled as a nutritional source depending on the sulfur and nitrogen contents of the medium [42]. Hence, the reason for the accumulated SAM when only xylose is available is probably sulfur and nitrogen starvation. Alternatively, the accumulation of SAM during xylose fermentation may be related to deficiencies in ergosterol biosynthesis [43], since a DNA microarray analysis of MA-R4 has revealed that many genes encoding proteins involved in ergosterol biosynthesis have significantly lower expression levels when cultivated with xylose alone than when cultivated with glucose alone (Matsushika et al. unpublished data). Thus, we hypothesize that the accumulation of polyamines, including SPD, was caused by high concentrations of SAM. It should be emphasized that this study represents the first showing that the fermentation of xylose leads to a drastic change in the polyamine metabolic pathway.

### Conclusions

Using a CE-TOFMS-based approach, our results successfully demonstrated that different carbon substrates, including glucose and xylose as single and mixed feed in fermentation medium, widely affected the dynamic metabolism of MA-R4. As a consequence, (i) when xylose replaced glucose as a substrate, almost all compounds from upper glycolysis and the PPP were of relatively high levels, while those of the lower glycolytic metabolites were significantly decreased; (ii) many amino acids and the energy charge tended to decrease when xylose instead of glucose was metabolized; (iii) the TCA cycle metabolite CIT and the aromatic amino acids were highly accumulated after fermentation with xylose alone; and (iv) fermentation with xylose alone led to the accumulation of SAM and its product SPD. These results suggest that low carbon flux through glycolysis from the PPP is one of the biggest factors restricting xylose utilization, and that carbon and energy starvation conditions are normal in MA-R4 during fermentation with xylose. Therefore, CE-TOFMS-based metabolomics is a powerful tool to understand how the lack of efficiency with which recombinant xylose-utilizing *S*. *cerevisiae* strains ferment xylose influences the yeast metabolome.
